# Evaluation of propofol anesthesia in morbidly obese children and adolescents

**DOI:** 10.1186/1471-2253-13-8

**Published:** 2013-04-21

**Authors:** Vidya Chidambaran, Senthilkumar Sadhasivam, Jeroen Diepstraten, Hope Esslinger, Shareen Cox, Beverly M Schnell, Paul Samuels, Thomas Inge, Alexander A Vinks, Catherijne A Knibbe

**Affiliations:** 1 Department of Anesthesia and Paediatrics, Cincinnati Children’s Hospital Medical Center, 3333 Burnet Ave, MLC 2001, Cincinnati, OH, 45229, USA; 2 Division of Pharmacology, Leiden/Amsterdam Center for Drug Research, Leiden, Netherlands; 3 Department of Anesthesia, Cincinnati Children’s Hospital Medical Center, Cincinnati, OH, USA; 4 Division of Clinical Pharmacology, Cincinnati Children’s Hospital Medical Center, Cincinnati, OH, USA; 5 Division of Biostatistics and Epidemiology, Cincinnati Children’s Hospital Medical Center, Cincinnati, OH, USA; 6 Division of Paediatric Surgery, Cincinnati Children’s Hospital Medical Center, Cincinnati, OH, USA; 7 Division of Clinical Pharmacology and Department of Paediatrics, Cincinnati Childrens Hospital Medical Center, Cincinnati, OH, USA; 8 University of Cincinnati, Cincinnati, OH, USA; 9 Department of Clinical Pharmacy, St. Antonius Hospital, Nieuwegein, Netherlands

**Keywords:** Morbidly obese, Bariatric, Propofol, Total intravenous anesthesia, Bispectral index, Anesthetic depth, Pediatric, Adolescents

## Abstract

**Background:**

Poor characterization of propofol pharmacokinetics and pharmacodynamics in the morbidly obese (MO) pediatric population poses dosing challenges. This study was conducted to evaluate propofol total intravenous anesthesia (TIVA) in this population.

**Methods:**

After IRB approval, a prospective study was conducted in 20 MO children and adolescents undergoing laparoscopic surgery under clinically titrated propofol TIVA. Propofol doses/infusion rates, hemodynamic variables, times to induction and emergence, and postoperative occurrence of respiratory adverse events (RAE) were recorded, along with intraoperative blinded Bispectral Index/BIS and postoperative Ramsay sedation scores (RSS). Study subjects completed awareness questionnaires on postoperative days 1 and 3. Propofol concentrations were obtained at predetermined intra- and post-operative time points.

**Results:**

Study subjects ranged 9 – 18 years (age) and 97 - 99.9% (BMI for age percentiles). Average percentage variability of hemodynamic parameters from baseline was ≈ 20%. Patients had consistently below target BIS values (BIS < 40 for >90% of maintenance phase), delayed emergence (25.8 ± 22 minutes), increased somnolence (RSS ≥ 4) in the first 30 minutes of recovery from anesthesia and 30% incidence of postoperative RAE, the odds for which increased by 14% per unit increase in BMI (p ≤ 0.05). Mean propofol concentration was 6.2 mg/L during maintenance and 1.8 mg/L during emergence from anesthesia.

**Conclusions:**

Our findings indicate clinical overestimation of propofol requirements and highlight the challenges of clinically titrated propofol TIVA in MO adolescents. In this setting, it may be advantageous to titrate propofol to targeted BIS levels until more accurate weight-appropriate dosing regimens are developed, to minimize relative overdosing and its consequences.

## Background

Propofol is commonly used for total intravenous anesthesia (TIVA) due to its characteristic ease of titration, rapid onset and offset of action, reduced incidence of postoperative nausea/vomiting [1] and emergence agitation [2]. In the morbidly obese (MO) paediatric population, despite propofol’s desirable characteristics, appropriate drug administration is complicated by numerous anatomic and physiological factors that accompany obesity, including increases in total body mass, blood volume, cardiac output and regional blood flow [3]. Inavailability of evidence-based clinical guidelines and an adequate dosing scalar for individualized propofol dosing in MO children and adolescents could adversely impact the quality of TIVA administered to these patients [4].

Recent evidence has highlighted drug dosing issues in obese adults raising concerns at both extremes of drug administration: inadequate anesthesia resulting in intra-operative awareness due to under-dosing propofol [5] and excessive anesthetic administration, resulting in organ hypoperfusion and low processed electroencephalographic index values which could be associated with poor outcomes [6-9]. Although the Bispectral Index/BIS monitor provides quantifiable and continuous assessment of propofol cortical effects in children and adolescents [10-12], it is a common to practice TIVA with propofol in children without BIS monitoring. In this descriptive study in a cohort of MO paediatric patients, we evaluated the effects of propofol TIVA on perioperative outcomes.

## Methods

A prospective study was conducted in MO children and adolescents between July 2009 and July 2010. The study protocol was approved by Cincinnati Children’s Hospital institutional review board and written informed assent / consent was obtained from all participants and/or their guardians as appropriate.

### Study subjects

Inclusion criteria: 1) Males and females between the ages of 5 and 18 years, 2) Body Mass Index (BMI) for age > 95^th^ percentile {> 95^th^ percentile (obese), >99^th^ percentile (MO) [13]}; 3) Patients undergoing elective surgery scheduled for a duration of at least 60 minutes.

Exclusion criteria: 1) Severe developmental delay, 2) Known cardiac anomaly, neurological, renal or hepatic disorders, 3) Known allergy to propofol, 4) Skin condition which would preclude placement of BIS sensor on the forehead.

### Study protocol

The patient was brought to the operating room, electrocardiograph, non-invasive blood pressure and pulse oximeter were applied, and an intravenous catheter was established. Before or immediately after induction, an age and head-size appropriate disposable BIS sensor® XP, (Aspect Medical Systems, Norwood, MA) was placed on each patient’s forehead and connected to the BIS monitor. The BIS monitor screen was covered throughout the procedure to blind the anesthesia personnel to the BIS score and trend screen. Anesthesia was induced with propofol at a standardized infusion rate of 1000 μg.kg^-1^.min^-1^ after intravenous injection of lidocaine 30 mg. Infusion rates were based on adjusted body weight (ABW) which was calculated using total body weight (TBW) and ideal bodyweight (IBW), as described by Servin *et. al*. [14], substituting 22 kg/m^2^ as Ideal BMI (in Servin’s formula) with 50^th^ percentile BMI for age and gender, obtained from Centers for Disease Control and Prevention, National Center for Health Statistics growth charts, United States. (http://www.cdc.gov/growthcharts/. May 30, 2000).

(1) $$\mathrm{ABW}=\mathrm{IBW}+0.4^{*}\left(\mathrm{TBW}-\mathrm{IBW}\right)$$

(2) $$\mathrm{IBW}=\mathrm{IdealBM}I^{*}{\left\{\mathrm{Height}\left(\mathrm{meter}\right)\right\}}^{2}$$

Patients were asked to count, or called repeatedly in a normal voice until the induction end-point of loss of verbal contact; this was recorded as ‘time to induction’. Succinylcholine was administered and the trachea was intubated with an appropriate cuffed endotracheal tube. Anesthesia was maintained with propofol infusion. Vecuronium was titrated to Train-of-Four response (goal: one of four twitches). The induction dose of propofol was followed by propofol infusion at a rate of 250-350 μg/kg/min for 10 minutes and titrated in 25-50 μg/kg/min steps (reduced to prevent drop in systolic arterial blood pressure and heart rate below 30% of baseline values and titrated up when greater than 30% increase in heart rate or blood pressure occurred in the absence of new painful stimuli). Propofol was infused using calibrated pumps with internal memory and downloading capability. This allowed all real-time rates and rate changes to be recorded, including start and stop time of propofol dosing, propofol infusion rates, and propofol dose adjustments. Fentanyl 50-100 μg was administered after induction and 50 μg doses were administered in case of inadequate analgesia (defined as increase in heart rate and/or blood pressure above 30% of baseline with surgical incision or manipulation). When inadequate anesthesia or analgesia was not considered to be the reason for increase in blood pressure or heart rate, medications to correct hemodynamics were administered. The propofol infusion was decreased by 50% about 15 minutes before conclusion of surgery and discontinued when skin sutures were being placed. Muscle relaxants were reversed and once the patient was breathing, morphine/hydromorphone was dosed incrementally towards the end of the surgery, titrated to respiratory rate of 14-16 breaths per minute. After clinical confirmation of reversal, the trachea was extubated awake. Patients were then transferred to the recovery area (PACU) and followed until they achieved PACU discharge criteria.

### Demographics

Patient demographics, age, gender, weight (TBW) and height were collected. After computing the BMI, IBW and ABW were calculated according to equations 1 and 2. Ideal BMI in Equation 2 is defined as the 50^th^ percentile values from age and sex – specific BMI for age charts at http://www.cdc.gov. A calculator available at http://www.bcm.edu/cnrc/bodycomp/bmiz2.html was used to calculate BMI for age percentiles. Lean body mass (LBM) was calculated using the formula described by Peters *et. al.* by first estimating Extracellular Volume (ECV) from weight and height [15] according to the following equation.

$$\mathrm{estimatedLBM}=3.8^{*}\mathrm{estimatedECV}$$

### Propofol and opioid doses

Posthoc calculation of induction dose required to achieve loss of verbal contact was performed by multipying the rate of infusion and time taken to reach the end-point. Means and SD of propofol infusion rates during maintenance were analyzed from pooled data. Propofol maintenance infusion rates were plotted against BIS values and time since start of propofol infusion. Infusion rates of eight patients corresponding to BIS values of 40-60 were then analyzed to derive mean and SD. Hourly opioid use as fentanyl equivalent doses were calculated, based on an equivalence of morphine 10 mg = 2 mg hydromorphone = 100 μg fentanyl.

### Hemodynamics

Clinical data including mean, systolic, diastolic blood pressure (MAP, SBP and DBP respectively) and heart rate/HR were recorded electronically every 5 minutes intraoperatively. For each of the measured hemodynamic parameters, percentage difference from baseline (value recorded 5 minutes before propofol induction) was calculated according to the following equation.

$$\mathrm{\%Difference}=100*\left(\frac{\mathrm{Value}-\mathrm{Baseline}}{\mathrm{Baseline}}\right)$$

### BIS

BIS data were transferred electronically to a computerized record in one-second increments. This included the date and time of BIS data collection, minimum and maximum BIS values, average Signal Quality Index (SQI) and average electromyography (EMG). The smoothing rate of the BIS monitor was set at 15 seconds. Evaluable BIS values were defined as those with Signal Quality Index > 70.

### Blood sampling and propofol analysis

Blood samples (1.0 ml) were obtained from a dedicated intravenous catheter placed in the upper extremity contralateral to the propofol infusion site. Samples were obtained at baseline prior to the start of propofol, approximately 15, 30, 45, 60, 120, 180, 240 minutes after the start of the propofol infusion, at 5 and 20 minutes after dose adjustment, just before discontinuation of the propofol infusion and at 5, 10, 15, 30, 45 and 120 minutes after termination of the infusion. Whole-blood samples for propofol analysis were stored at 4°C until analysis (within 1 month) by high-performance liquid chromatography with fluorescence detection. The coefficients of variation for the intra-assay and interassay precision over the concentration range from 0.05 to 5.0 mg.l^-1^ were less than 4.5% and 7.1% respectively. The lower limit of quantification was 0.05 mg.l^-1^[16].

### Ramsay sedation scores

Ramsay Sedation Scores (RSS) were recorded post-operatively about every 10 minutes for the first 30 minutes and thereafter every 30 minutes while in the PACU [17].

### Other clinical data

‘*Time to eye opening’*, defined as the time from cessation of propofol infusion to eye opening on verbal command, was noted. *Respiratory adverse events (RAE)* defined as airway obstruction requiring airway manipulation, episodes of desaturation (< 90%) and/or need for oxygen for >120 minutes in the immediate postoperative period were also recorded. On postoperative day 1 and 3, patients were evaluated using the *Structured Awareness Screening Interview* created by Davidson *et. al*. [18].

### Statistical analysis

GraphPad Prism 5 software (GraphPad Software Inc., La Jolla, CA) was used to generate descriptive statistics (mean, standard deviation, median and range for continuous variables and frequencies for categorical variables). Linear, quadratic and cubic trends were tested to detect correlation of weight scalars (TBW, ABW and LBM) with induction dose, in addition to calculation of root mean square errors (MSE) and the regression lines fitted. SAS software © (SAS version 9.2, Cary, North Carolina) was used to perform logistic regression between occurrence of respiratory adverse events and explanatory variables (TBW, IBW, ABW, BMI, propofol amount and duration of propofol infusion) to detect two-tailed p values with a 95% Confidence Intervals (CI).

## Results

### Demographics

Patient and surgical characteristics are presented in Table 1. Of 23 patients enrolled, 20 were fully evaluable. One patient withdrew shortly before the procedure (no samples); and two patients were excluded because of difficulty obtaining blood samples from existing intravenous lines. 19 patients met criteria for morbid obesity.

**Table 1:** Patient characteristics

**Characteristic**	**Mean**	**SD**	**Range**
**Age (years)**	15.8	2.2	9 - 18
**Body weight (kg)**	125.1	29.1	69.6 - 184
**Ideal Body Weight (kg)**†	55.5	9.6	34.3 - 74
**Body MASS INDEX/BMI (kg/m**^**2**^**)**	45.6	9.2	31.3 – 62.9
**BMI for age percentiles**	99.4	0.7	97-99.9
**Lean Body Mass**††	74.6	13.6	46.7-98.4
**Sex (F - M)**	12 – 8 (60%-40%)		
**Co-morbidities** (n = number of patients with condition)	Insulin Resistance (n = 6) Mild Obstructive Sleep Apnea (n = 7); Hypertension (n = 5); Diabetes (n = 1); Asthma (n = 3); Dyslipidemia (n = 2); Gastroesophageal Reflux (n = 4); Depression/Anxiety (n = 4).		
**Surgeries** (n = number of patients who underwent the procedure)	Bariatric - Laparoscopic Gastric bypass or Sleeve Gastrectomy (n = 11), Laparoscopic cholecystectomy/appendectomy (n = 6), Orthopedic procedures on lower extremities (n = 3).		

Table 1: Characteristics of the 20 evaluable patients are presented as mean, standard deviation (SD) and range. Derived weight parameters are †Ideal Body Weight = Ideal BMI * {Height (meter)} ^2^ where Ideal BMI is defined as the 50^th^ percentile values from age and sex – specific BMI for age charts at http://www.cdc.gov. ¶A calculator available at the http://www.bcm.edu/cnrc/bodycomp/bmiz2.html was used to calculate the BMI for age percentiles. ††Calculated using Peters et. al. Formula (see Methods).

### Propofol and opioid doses

**Figure 1:**
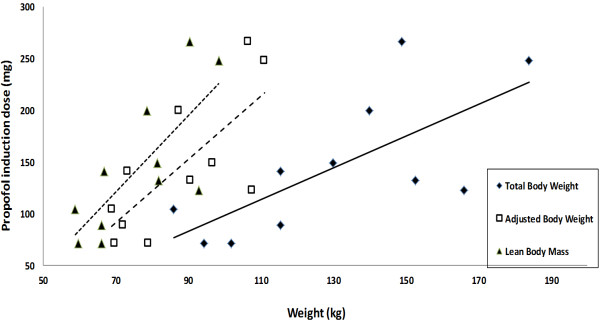
**Linear regression of propofol induction dose to weight scalars.** Linear regression trendlines for correlation of posthoc calculated induction dose of propofol (titrated to loss of verbal contact) with weight scalars are shown. The correlation coefficients, Root Mean Square Errors (Root MSE) and p-values for the correlations were found to be R^2^ = 0.58, Root MSE = 45.92, p = 0.0068 for Lean Body Mass (LBM), R^2^ = 0.54, Root MSE = 47.82, p = 0.01 for Adjusted Body Weight (ABW) and R^2^ = 0.5, Root MSE = 49.61, p = 0.0143 for Total Body Weight (TBW).

**Figure 2:**
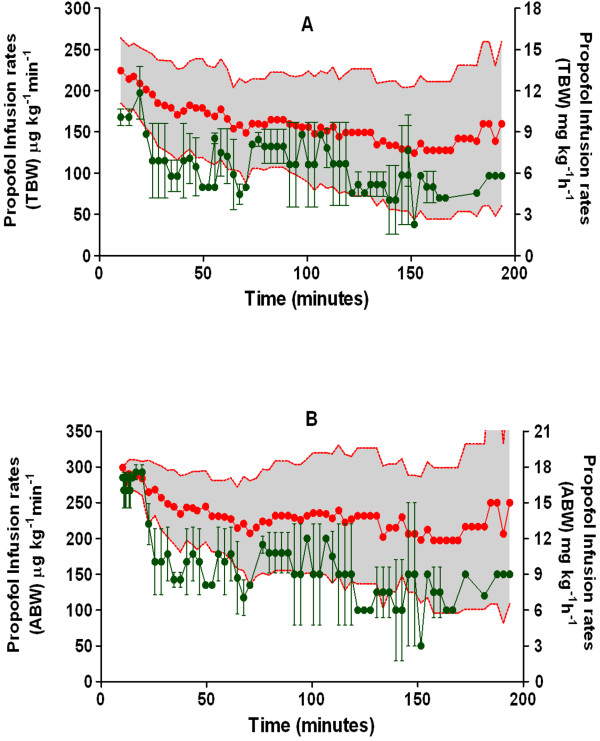
**Maintenance propofol infusion rates.** Data analysis of propofol infusion rates used during the maintenance phase (in μg kg^-1^ h^-1^ on the left Y-axis and mg kg^-1^ h^-1^ on the right Y-axis) based on total body weight (TBW) and adjusted body weight (ABW) are depicted in (**A**) and (**B**) respectively. The *red solid circles* and the *grey shaded area* within the error bands (*red dotted lines*) represent the means and SD of actual administered infusion rates over time, while the *green dots and vertical lines* represent the means and SD of infusion rates corresponding to BIS values of 40-60.

Hourly propofol and fentanyl equivalent doses, as well as calculated induction doses, are presented in Table 2. Data from four patients were excluded from the calculation of propofol induction dose, due to the use of boluses and protocol deviations from standardized infusion for induction. Only linear regression of induction dose and weight scalars was found to be significant. They are depicted in Figure 1. LBM were the most highly correlated to the induction dose with least root MSE. Means and standard deviations (SD) of administered propofol maintenance rates and rates corresponding to BIS 40-60 based on TBW (Figure 2A) and ABW (Figure 2B) are presented. Number of paired observations for the latter calculation from data of 8 patients, was 116; evaluable BIS values 0.5-3 minutes apart were included to maximize available data in that BIS range. Infusion rates administered were consistently higher than those that were found to correlate with BIS 40-60.

**Table 2:** Propofol dosing and clinical parameters

**Dosing characteristic**	**Mean**	**SD**	**Range**
Duration of propofol infusion (min)	135	61	41 - 291
Total amount of propofol (mg)	3244	2205	962 - 10507
Propofol dose in mg.kg^-1^ h^-1^	11.5	3	6.9-17.8
Induction dose (mg.kg^-1^ TBW)	1.3	0.5	0.7 - 2.1
Induction dose (mg. kg^-1^ ABW)	1.9	0.8	0.9 - 3.2
Fentanyl equivalent doses in μg.h^-1^	175	70	50-300
**Clinical parameters**			
Time to induction (min)	1.5	0.5	0.92 - 2.3
Time to eye opening (min)	25.8	22.6	1.5 - 93.7
Incidence of adverse respiratory events	6/20 (30%)		
Incidence of awareness	0/20		

Table 2: Means, standard deviations (SD) and range of propofol and opioid dosing characteristics received by study subjects, and measured clinical outcomes are tabulated. Induction dose is the calculated dose to achieve clinical end point and is expressed per total body weight (TBW) and adjusted body weight (ABW).

### Hemodynamics

**Figure 3:**
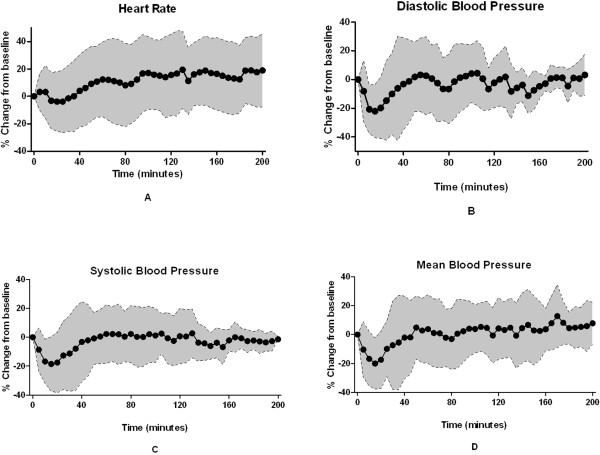
**Variability of hemodynamic parameters over time during propofol anesthesia.** In this figure, time profiles of variability of heart rate (**A**), diastolic blood pressure (DBP) (**B**), systolic blood pressure (SBP) (**C**) and mean blood pressure (MAP) (**D**) are presented as the mean (*black solid circles*) and standard deviation (SD) {*grey shaded area* between error bands (*black dotted lines*)} of the % change from baseline for the stated parameter, plotted every 5 minutes during 200 minutes of propofol anesthesia. The first dot on the timeline represents the start of propofol induction (baseline) and hence % variability is 0%.

Figure 3 shows the mean and SD of the percentage difference from baseline for HR (1A), DBP (1B), SBP (1C) and MAP (1D) from 5 minutes prior to start of propofol to 200 minutes of propofol anesthesia. SBP, MAP and DBP values declined by about 20%, reaching a nadir at about 15 minutes after induction and returning to baseline values in 30-40 minutes. Overall, average percentage variability from baseline was 20%. Labetolol was used in one patient and the data from this patient were excluded from this analysis.

### Intraoperative propofol plasma concentrations and BIS scores

**Figure 4:**
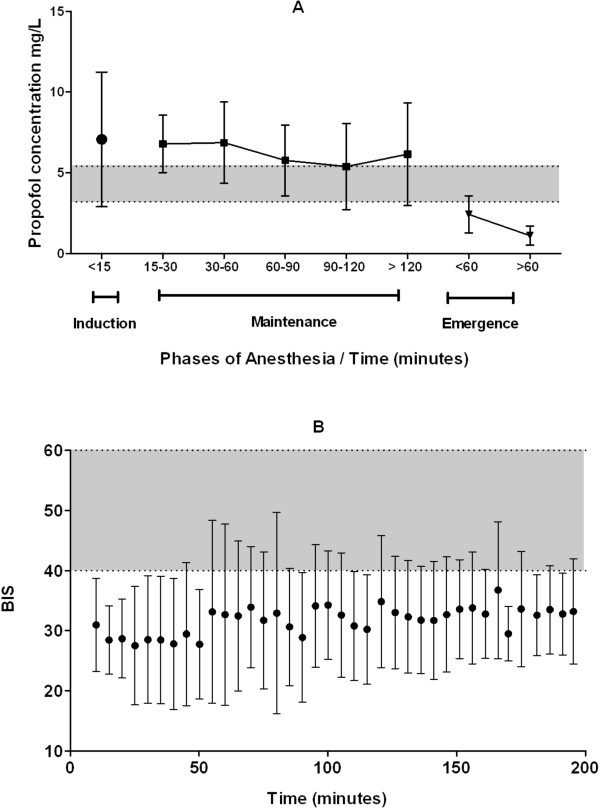
**Summary of propofol concentrations during different phases of propofol anesthesia and BIS values over time.** (**A**) represents the means (*black solid circles for induction phase, squares for maintenance and inverted triangles for emergence phase*) and standard deviations (SD) (*black vertical lines*) of propofol concentrations during different phases of anesthesia; The propofol concentration during the induction phase (first 15 minutes) was 7.0 ± 4.1 mg.l^-1^ (n = 16). The mean (SD)(number of samples) for propofol concentrations collected during 15-30, 30-60, 60-90, 90-120 and >120 minute time intervals of maintenance anesthesia were 6.8(1.8)(26), 6.9(2.5)(41), 5.8(2.2)(36), 5.4(2.7)(15) and 6.1(3.2)(29) mg.l^-1^ respectively. (**B**) shows the means (*black solid circles*) and SD (*black vertical lines*) of blinded BIS values during 10-200 minutes of maintenance phase of propofol anesthesia. *Grey shaded areas* depict range of propofol concentrations reported to be associated with BIS 50 in children (A) (3.2-5.4 mg.l-1: Riguozzo *et. al*, 2010) and BIS values generally considered to infer adequate depth of anesthesia (B) (46-60) for this population.

An average of 14 venous samples was collected per patient. In Figure 4A, the means ± SD of propofol concentrations during different phases of anesthesia are shown. BIS data were not retrievable for one patient due to software malfunction. Figure 4B shows the means and SD of BIS values recorded every 5 minutes during the maintenance phase of propofol anesthesia (excluding 1^st^ ten minutes after induction and the last ten minutes of emergence for every patient). It is noteworthy that the BIS values were in the range of 20-40 for 89.4% and less than 20 for another 3.9% of the maintenance phase. Nineteen of twenty patients had BIS levels less than 40 for at least 20 minutes of maintenance.

### Postoperative propofol concentrations and RSS

Propofol concentrations declined to 2.4(1.2) mg.l^-1^ during the first hour of emergence and remained at 1.1(0.6) mg.l^-1^ during the second hour after the discontinuation of propofol infusion (Figure 4A). Ramsay sedation scores > 4 were present up to 30 minutes after arrival to the PACU, which indicates deep sedation. Spearman Rank correlation between RSS and the propofol concentrations was found to be 0.65 (p < 0.0001).

### Other clinical data

Time to induction, eye opening and incidence of awareness are presented in Table 2. Six patients had RAE in the PACU – one patient had airway obstruction requiring airway manipulation to correct mild hypoxemia, and five others had an extended requirement for oxygen (>120 minutes) to maintain saturation > 90%. BMI was significantly associated with the likelihood of having an adverse respiratory event in the PACU (p = 0.05). For every unit increase in BMI, there was a corresponding increase of 14% in the odds of having an adverse respiratory event.

## Discussion

TIVA with propofol in MO pediatric patients can be challenging in the absence of weight and dosing guidelines.We evaluated the clinical response to propofol anesthesia in this population.

While hemodynamic parameters during propofol TIVA were largely unchanged, BIS values for MO adolescents were below 40 for 93% of the maintenance phase. We believe that the increased anesthetic depth was a result of clinical overestimation of propofol requirements. Although our study did not have a BIS control group, our findings that MO adolescents undergoing clinically titrated propofol TIVA received high propofol doses, is in accordance with what has been reported in obese adults. Gaszynski *et. al*. demonstrated that obese adults undergoing clinically titrated propofol TIVA without BIS monitoring received higher propofol infusions (10 vs. 5.8 mg.kg-1/h), consumed more total propofol (2012 ± 310 mg vs. 1210 ± 370 mg) and had longer awakening times [19], compared to those who were BIS monitored.

There are two other findings of significance. Firstly, prolonged emergence from anesthesia was observed in our study patients, with an average ‘time to eye opening’ of 25.9 ± 22.6 min, compared to 10.3 ± 5.4 minutes reported in non-obese children after clinically titrated propofol TIVA [19,20]. This was also reflected by deeper levels of sedation (RSS > 4) during the first 30 minutes in the PACU. Although there is some evidence for propofol accumulation and slow washout after continuous propofol infusions in MO adults [21], this has not been supported by clinical data in adults [14]. We believe the prolonged emergence is due to the high propofol doses our study subjects received (mean = 3244 mg or 11.5 mg kg^-1^ h^-1^), which positively correlated with the ‘time to eye-opening’ (p = 0.03). Secondly, we note a 30% incidence of RAE in the immediate postoperative period with a 14% increased risk of RAE for every unit increase in BMI. Increased risk of RAE after propofol TIVA in obese patients, is supported by Zoremba *et. al*.’s finding of excessive impairment of pulmonary function in obese adults, two hours after propofol anesthesia [22].

Despite the fact that in clinical settings, propofol is generally administered as a bolus for induction, we chose to use a standardized infusion method for induction. This allowed us to calculate an induction dose based on a clinical endpoint rather than an arbitrary weight-based dose. We noted a high correlation for induction dose to LBM (similar to findings of Ingrande *et. al*.) [23] and ABW which suggests that the dosing for induction be based on these scalars and not TBW. These findings need to be confirmed with large prospective studies and a formal pharmacokinetic-pharmacodynamic analysis. Pharmacokinetic analysis following this study has been completed and results have been published in an earlier report wherin TBW proved to be the most significant determinant for clearance, while no predictive covariates for volume of distribution were identified [24]. Our infusion regimen was based on ABW as Servin *et. al*. had used this weight in morbidly obese adults without evidence of propofol accumulation [14]. Our finding that an average infusion rate of 7 mg kg^-1^ h^-1^ TBW during 20-90 minutes of propofol maintenance phase correlates with a BIS of 40-60 (Figure 2A), is higher than the recommended rate of 4.6 to 6 mg kg^-1^ h^-1^ TBW to maintain BIS of 50 in obese adults during the same time period [14,25]. Considering that concentrations of 4.3 ± 1.1 mg.l^-1^ in non-obese children [11] and 3-4 mg.l^-1^ in obese adults, have been reported to correlate with a BIS of 50 [26], our findings of higher propofol concentrations during maintenance of anesthesia and corresponding lower BIS values suggests that clinical titration of propofol anesthesia in MO adolescents is not optimal.

Ramsay sedation scores were used to assess sedation in the postoperative period. We used a single non-anesthesia observer to rate RSS in all study subjects to limit inter-observer variance. However, caution is required in interpreting correlation of propofol concentrations with RSS as these sedation scores reflect the combination of propofol and opioid effects. We also note that the observational study design allowing clinical titration of propofol doses prevented standardization of dosages. Although dosing of propofol could be affected by differences in opioid doses, it has been reported to not affect the relation between propofol concentrations and BIS [27]. Hence, the lack of standardization of opioid doses would likely not affect our conclusions. Finally, our premise that BIS values below 40 represent very ‘deep’ anesthesia is debatable, but there is no evidence to the contrary, as none of our patients suffered any awareness. The other dilemma concerning the risks associated with excessive anesthesia dosing is still unresolved [28].

## Conclusion

In conclusion, this study presents a detailed descriptive analysis of propofol anesthesia in MO adolescents. Although BIS has been found to improve clinically important outcomes in children undergoing inhalation anesthesia [29], it is not a standard monitor in paediatric anesthetic practice. In MO adults, La Colla *et. al*. concluded that it is advisable to administer propofol to MO patients by titration to targeted processed-EEG values [30]. Our findings suggest that in the absence of evidence based dosing guidelines for propofol administration in this MO paediatric population, use of only clinical parameters to dose TIVA with propofol can result in excessive depth of anesthesia. In this setting, BIS monitoring provides anesthesiologists information about real time trend of anesthetic depth and helps prevent excessive propofol administration and associated negative consequences. Our findings also emphasize the need for improved propofol dosing guidelines and monitoring during TIVA in MO adolescents to minimize relative overdosing and its negative consequences.

## Abbreviations

BIS: Bispectral index; MO: Morbidly obese; TIVA: Total Intravenous anesthesia; RSS: Ramsay sedation score

## Competing interests

The authors declare that they have no competing interests.

## Authors’ contributions

VC was involved in design, conduct of the study, analysis of the data, and manuscript preparation, SS helped design, conduct the study, and write the manuscript, JD helped design the study and write the manuscript, HE was involved in conduct of the study, SC helped conduct the study and analyze propofol, BS analyzed the data, PS participated in the conduct of the study and manuscript writing, TI helped conduct of the study, AAV and CAK were involved in designing the study, analysis of the data, and preparation of the manuscript. All authors read and approved the final manuscript.

## Pre-publication history

The pre-publication history for this paper can be accessed here:

http://www.biomedcentral.com/1471-2253/13/8/prepub
